# Bioproduction of L‐piperazic acid in gram scale using *Aureobasidium* *melanogenum*

**DOI:** 10.1111/1751-7915.13838

**Published:** 2021-06-03

**Authors:** Cuncui Kong, Zhuangzhuang Wang, Guanglei Liu, Zhenming Chi, Rodrigo Ledesma‐Amaro, Zhe Chi

**Affiliations:** ^1^ College of Marine Life Sciences Ocean University of China No.5 Yushan Road Qingdao 266003 China; ^2^ Pilot National Laboratory for Marine Science and Technology No.1 Wenhai Road Qingdao 266237 China; ^3^ Department of Bioengineering Imperial College London London SW7 2AZ UK

## Abstract

Currently, piperazic acid is chemically synthesized using ecologically unfriendly processes. Microbial synthesis from glucose is an attractive alternative to chemical synthesis. In this study, we report the production of L‐piperazic acid via microbial fermentation with the first engineered fungal strain of *Aureobasidium melanogenum*; this strain was constructed by chassis development, genetic element reconstitution and optimization, synthetic rewiring and constitutive genetic circuit reconstitution, to build a robust L‐piperazic acid synthetic cascade. These genetic modifications enable *A*. *melanogenum* to directly convert glucose to L‐piperazic acid without relying on the use of either chemically synthesized precursors or harsh conditions. This bio‐based process overcomes the shortcomings of the conventional synthesis routes. The ultimately engineered strain is a very high‐efficient cell factory that can excrete 1.12 ± 0.05 g l^‐1^ of L‐piperazic acid after a 120‐h 10.0‐l fed‐batch fermentation; this is the highest titre of L‐piperazic acid reported using a microbial cell factory.

## Graphical abstract

L‐piperazic acid via microbial fermentation with the first engineered fungal strain of *Aureobasidium* *melanogenum* was achieved. This strain was constructed by chassis development, genetic element reconstitution and optimization, synthetic rewiring, and constitutive genetic circuit reconstitution to build a robust L‐piperazic acid synthetic cascade. The ultimately engineered strain has the highest titer of L‐piperazic acid of 1.12 ± 0.05 g l^‐1^ ever reported.

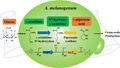

## Introduction

Piperazic acid (Piz; PubChem CID: 2762538), a cyclic hydrazine and a non‐proteinogenic amino acid, has been found in over 140 molecules of natural products, many of which exhibit promising biological activities (Ciufolini and Xi, 1998; Morgan *et al*., 2019): antrimycins (Shimada *et al*., 1981) and svetamycins (Dardic *et al*., 2017) as tuberculostatic agents, kutznerides (Broberg *et al*., 2006) as antimicrobial agents and luzopeptins as anti‐human immunodeficiency viral agents (Ohkuma *et al*., 1980). Moreover, Piz has been shown to be able to induce β‐turns in peptides (Ciufolini and Xi, 1998). Therefore, Piz has gained increasing popularity in natural L‐product chemistry, pharmaceutical research and peptide/protein engineering (Morgan *et al*., 2019). Of note, it is an important intermediate building block in the chemical synthesis of the first‐line antihypertensive drug cilazapril (Adams *et al*., 1988; Attwood, 1989) and interleukin‐1β converting enzyme (ICE) inhibitors (Robidoux *et al*., 2001), highlighting its importance in practical pharmaceutical enterprises.

**Fig. 1:**
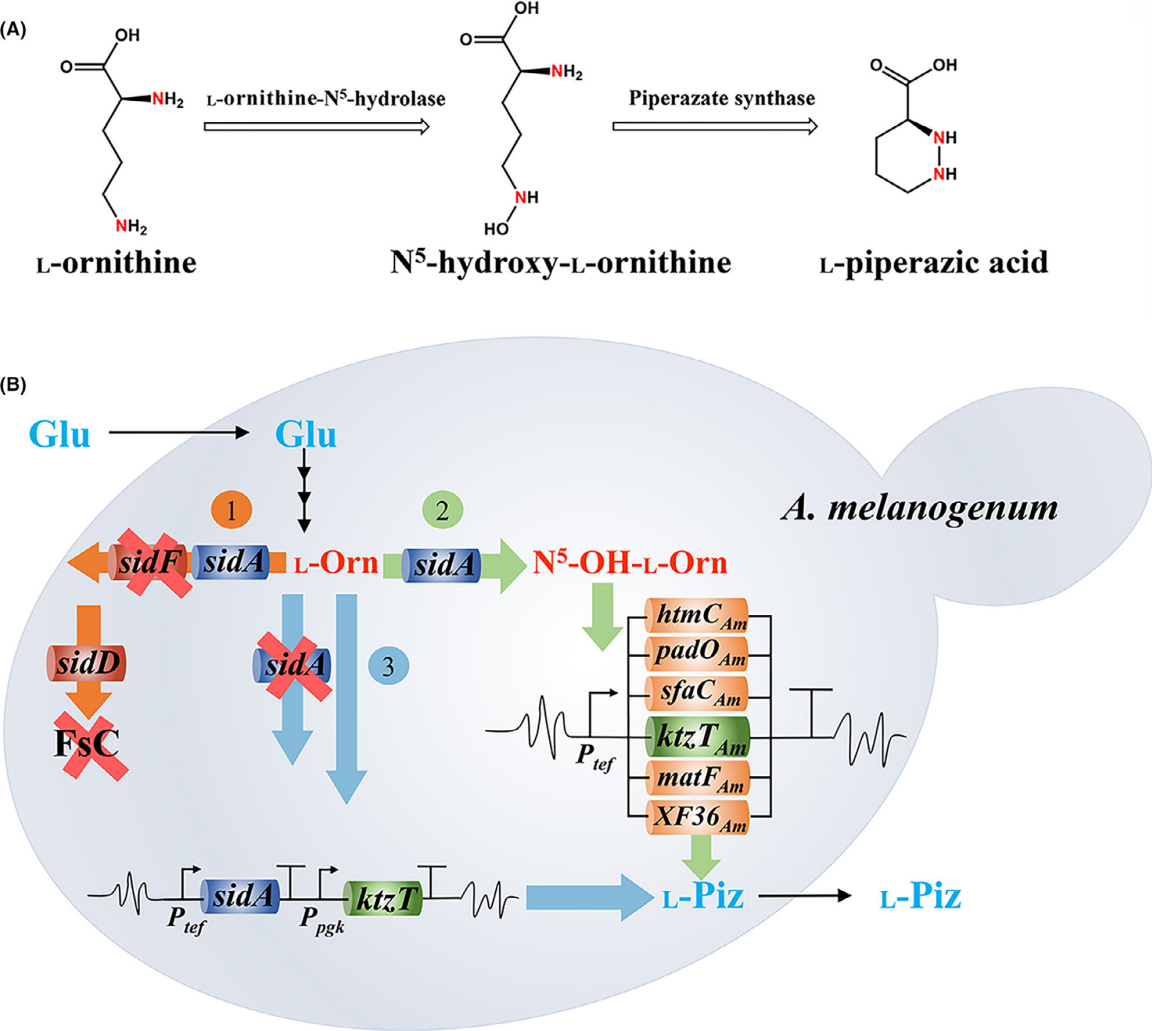
(A) Biosynthetic pathway of L‐piperazic acid from L‐ornithine precursor. (B) Bioengineering *A*. *melanogenum* strain for the production of L‐piperazic acid. Glu, glucose; L‐Orn, L‐ornithine; N^5^‐OH‐L‐Orn, N^5^‐hydroxy‐L‐ornithine; L‐Piz, L‐piperazic acid. *sidA*, L‐ornithine‐N^5^‐hydroxylase gene; *sidF*, anhydromevalonyl‐CoA transferase gene; *sidD*, FsC synthase gene; *htmC_Am_*, *pad*O*_Am_*, *sfaC_Am_*, *kztT_Am_*, *matF_Am_* and *XF36_Am_*, six piperazate synthase gene variants. *Ptef*, promoter of gene encoding transcription elongation factor 1 from *A*. *melanogenum* HN6.2 strain; *Ppgk*, promoter of gene encoding phosphoglycerate kinase from *A*. *melanogenum* HN6.2 strain. The orange circle symbol of 1 and arrows 1 indicate the first module of genetic modification, so forth the light green module 2 and light blue module 3. Deleted gene and elimination of FsC siderophore are marked with a bold red cross. Six piperazate synthase genes were overexpressed in Module 2.

Thus far, different routes have been proposed for the chemical synthesis of Piz: de novo construction with hydrazine units (Ciufolini and Xi, 1998; Robidoux *et al*., 2001) and the Diels–Alder and aza‐Diels–Alder reactions (Kaname *et al*., 2009). However, these processes are complicated and not eco‐friendly. Therefore, new bio‐based green synthesis processes must be developed – enzymatic reactions or whole cell bioconversion. In 2017, a heme‐dependent piperazate synthase KtzT that is responsible for catalysing the conversion of N^5^‐hydroxy‐L‐ornithine (Neumann *et al*., 2012) (PubChem CID: 169671) into L‐Piz in the *Kutzneria* sp. 744 strain was identified (Du *et al*., 2017). Furthermore, in this strain, a flavin‐dependent L‐ornithine‐N^5^‐hydroxylase KtzI that converts L‐ornithine (PubChem CID: 6262) to N^5^‐hydroxy‐L‐ornithine (Neumann *et al*., 2012) was discovered. *ktzI* and *ktzT* homologs have been found in gene clusters in various bacteria that produce Piz‐containing molecules (Morgan *et al*., 2019). This indicates the existence of biosynthetic pathways of L‐Piz (Fig. 1A). Accordingly, some effort has been invested in the synthesis of L‐Piz via biological approaches rather than the conventional chemical approach. For example, the coupled use of recombinant KtzI and KtzT has been shown to catalyse the production of L‐Piz from L‐ornithine (Du *et al*., 2017). In another case, L‐Piz was synthesized from glucose using *Streptomyces* species via the reconstituted genetic cascade of *sfaB* (homolog of *ktzI*) and *sfa*C (homolog of *ktzT*) (Hu *et al*., 2019), but the obtained L‐Piz titre was extremely low, in the milligram scale. Therefore, these reports seldom possessed prospects of being applicable to industries, leaving this issue unresolved.

*Aureobasidium* spp. are biotechnologically significant yeast‐like fungi that have great potential in industrial transformation, thereof the species of *A. pullulans* and *A*. *melanogenum* have been used for the industrial production of pullulan (Chi *et al*., 2009 and Chi *et al*., 2016). We previously found that N^5^‐hydroxy‐L‐ornithine is a precursor for the produced hydroxamate siderophores in the yeast‐like fungus *Aureobasidium melanogenum*. This precursor is synthesized via the catalysis of SidA, a homolog of KtzI, using L‐ornithine as the substrate (Lu *et al*., 2019a,2019b). In addition, we developed an efficient genome editing system for *A*. *melanogenum* (Lu *et al*., 2019a,2019b; Zhang *et al*., 2019). These facts suggest that it is possible to set up a microbial cell factory for the heterologous production of Piz by introducing a piperazate synthase gene into the genome of *A*. *melanogenum*. Moreover, high yields of Piz can be obtained by modifying other metabolic pathways related to the biosynthesis of the precursors of Piz (Fig. 1A). In this study, we rewired the original metabolism of *A*. *melanogenum* so as to enable it to produce L‐Piz from feedstock without relying on any chemically synthesized precursor and requiring organic solvents or other special conditions. This study showcases a potential route that is alternative to the present chemical synthetic process, for the production of the valuable chemical block of L‐Piz by using a completely microbial approach.

## Results and discussion

### Elimination of siderophore biosynthesis to develop a chassis strain

To achieve high‐yield production of Piz in *A*. *melanogenum*, sufficient supply of its precursor should be guaranteed. As L‐ornithine is the starting material for Piz production (Fig. 1A), the *A*. *melanogenum* mutant strain DOLC19, which can accumulate an approximately 19‐fold higher content of intracellular L‐ornithine than that of its wild‐type strain HN6.2 owing to its ornithine carbamoyltransferase (OTC) deficiency (Lu *et al*., 2019a,2019b), was used as the precursor strain so as to satisfy such a sufficient supply of precursors for the biosynthesis of Piz.

Moreover, N^5^‐hydroxy‐L‐ornithine, the direct precursor for Piz, must be catalysed from L‐ornithine as its precursor (Fig. 1A). In *A*. *melanogenum*, siderophore biosynthesis is the only known metabolic pathway that uses N^5^‐hydroxy‐L‐ornithine as the precursor (Lu *et al*., 2019a,2019b). Previously, we reported that the biosynthesis of ferricrocin and hydroxyferricrocin siderophores in the recombined *A*. *melanogenum* strain DOLC19 was eliminated through the deletion of the L‐ornithine‐N^5^‐transacetylase gene *sidL* and the FC synthase gene *sidC* (Lu *et al*., 2019a,2019b); however, the biosynthesis of fusarinine C (FsC) and FsC‐derived fusarinine B (FsB) siderophores was still active. To completely block the consumption of N^5^‐hydroxy‐L‐ornithine through the biosynthesis of FsC and FsB in the DOLC19 strain, the first genetic manipulation was the deletion of the anhydromevalonyl‐CoA transferase gene *sidF*, which converts N^5^‐hydroxy‐L‐ornithine into FsC (Hu *et al*., 2019) (Fig. 1B, Module 1), yielding SidF‐null strain DF1. After the DF1 strain was cultivated in an iron‐depleted (modified sucrose peptone; MSP) medium (Lu *et al*., 2019a,2019b) for 120 h, high‐performance liquid chromatography (HPLC) analysis showed that none of the characteristic peaks of FsC and FsB siderophores produced by the DOLC19 strain (Fig. S1A) could be detected in the fermentation broth of the DF1 strain (Fig. S1B). Furthermore, the iodine oxidation approach also confirmed that no siderophores could be synthesized using the DF1 strain (Table S1), indicating that this strain completely lost its biosynthetic ability to form siderophores.

**Fig. 2:**
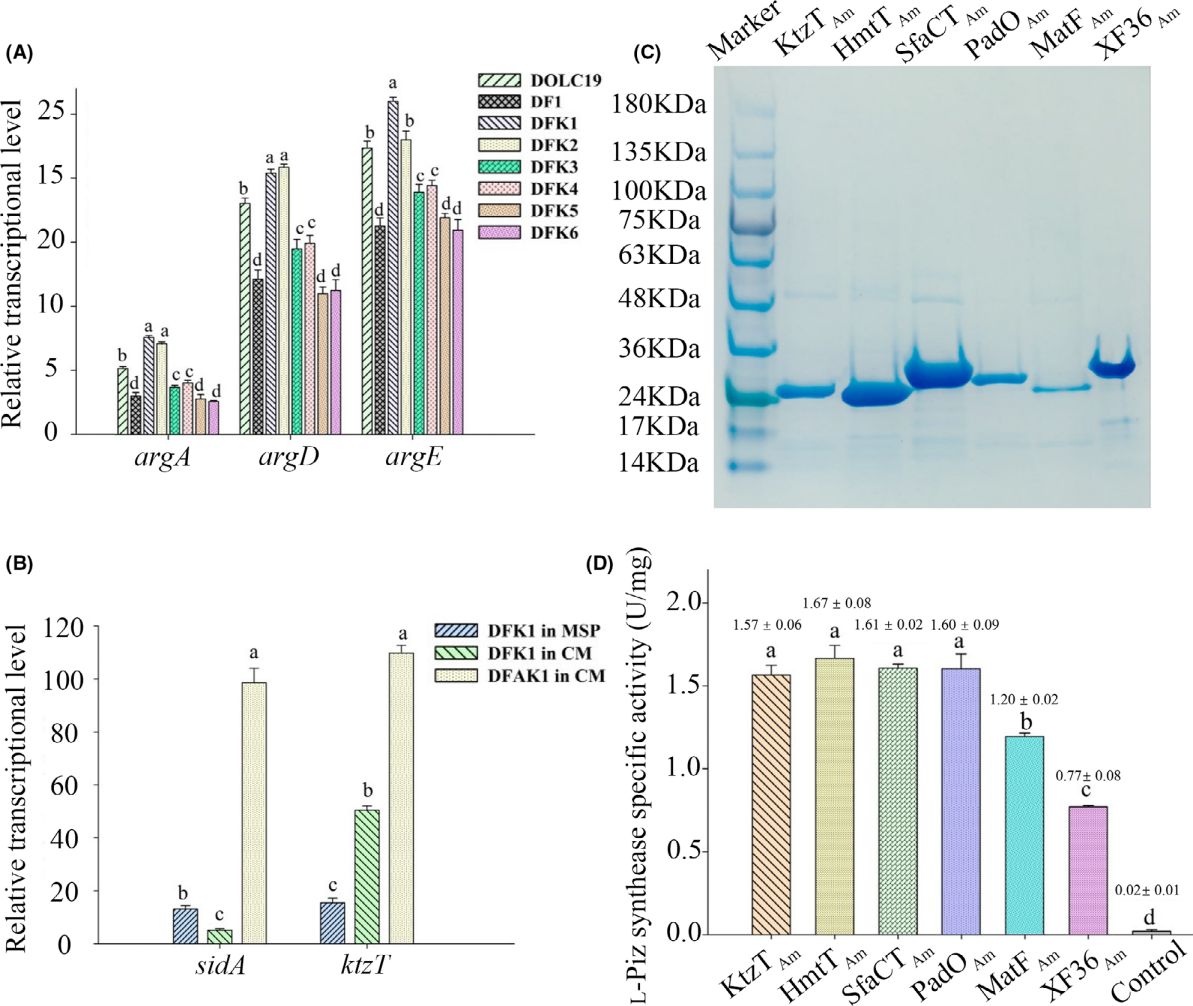
Relative transcriptional levels for the L‐ornithine synthetic genes in various engineered *A*.*melanogenum* strains in this study (A) and the L‐ornithine‐N^5^‐hydroxylase gene *sidA* and piperazate synthase gene *ktzT* in different strains under respective cultivating conditions with different iron availability (B); Purified piperazate synthases of water‐soluble fractions from *E. coli* recombination (C) and their specific enzyme activities (D). Data were presented as mean ± standard deviation, *n* = 3; abcd, data with different symbols had significant difference. *argA*, *N*‐acetylglutamate synthase; *argD*, *N*‐acetylornithine‐ aminotransferase; *argE*, *N*‐acetylornithine deacetylase.

It was determined that, albeit slightly decreased, there was no significant difference between the intracellular L‐ornithine content of the DF1 strain and that of the DOLC19 strain (Table S1), indicating that the elimination of siderophore biosynthesis does not impair L‐ornithine accumulation. Moreover, transcriptional analysis reflected that the L‐ornithine synthetic genes of *N*‐acetylglutamate synthase *argA*, *N*‐acetylornithine aminotransferase *argD* and *N*‐acetylornithine deacetylase *argE* (Lu *et al*., 2019a,2019b) in the DF1 strain exhibited downregulations as compared with the genes of those in the DOLC19 strain (Fig. 2A). Correspondingly, the intracellular content of N^5^‐hydroxy‐L‐ornithine decreased in the DF1 strain as compared to that in the DOLC19 strain (Fig. S2). Therefore, we postulated that the downregulation of these L‐ornithine synthetic genes could be ascribed to feedback inhibition (Beckmann *et al*., 2013) by N^5^‐hydroxy‐L‐ornithine that came from the SidF deficiency. Overall, these data demonstrated that the DF1 strain is a suitable chassis for Piz biosynthesis as it is likely enhanced in the precursor N^5^‐hydroxy‐L‐ornithine.

### Generation of L‐Piz using *A. melanogenum*

For catalysing the production of Piz in the developed *A*. *melanogenum* chassis, the codon‐optimized piperazate synthase gene variant *ktzT_Am_*, which was derived from the *ktzT* gene of the *Kutzneria* sp. 744 strain (Table S2), was introduced into the genome of the DF1 strain (Fig. 1B, Module 2), thereby producing the recombinant strain DFK1. Following cultivation in MSP medium for 120 h, the presence of Piz in the fermentation broth was first confirmed using HPLC and liquid chromatography–mass spectrometry (LC–MS) after precolumn derivatization with 9‐fluorenylmethoxycarbonyl‐chloride (Fmoc‐Cl). As a result, the retention time by HPLC (Fig. S1C) and the mass spectrum from LC–MS (*m/z* = 353.19, Fig. S3) of the product found was in close agreement with the results of the Fmoc‐Cl‐derivatized racemic D/L‐Piz standard and those reported in a previous study (Du, *et al*., 2017), thereby suggesting that the DFK1 strain was able to produce Piz.

To acquire a more convincing evidence about the structure of the produced compound, the Fmoc‐Cl‐derivatized product was subjected to preparative HPLC for purification (Fig. S4). Then, the purified Fmoc‐Piz was treated with NaN_3_ to eliminate the Fmoc moiety from the molecule(Chen *et al*., 2014); 10 mg of the obtained Fmoc‐eliminated product was analysed using nuclear magnetic resonance (NMR). The interpretation of ^1^H‐NMR and ^13^C‐NMR spectra (Fig. S5) confirmed this product was indeed Piz. Finally, the absolute configuration of this product was determined using Marfey’s method (Williams *et al*., 2011). The retention time for the 1‐fluoro‐2,4‐dinitrophenyl‐5‐L‐alanine amide (L‐FDAA) derivative of the fungal product (*m/z* = 383.09, Fig. S6) from HPLC matched that of the later elution of the racemic D/L‐Piz standard (Fig. S1D), which was previously specified to be L‐Piz (Williams *et al*., 2011). This was also consistent with the fact that L‐Piz was synthesized with the precursor of the natural L‐type ornithine via the use of biological enzymes in *A*. *melanogenum*. All of these results demonstrate that L‐Piz was successfully synthesized using the recombinant *A*. *melanogenum* DFK1 strain.

### Identification of the optimal piperazate synthase gene element

Screening for the best performing heterologous gene was important to optimize the bioconversion efficiency (Qin *et al*., 2015; Luo *et al*., 2019). Therefore, upon the successful biosynthesis of L‐Piz using A. melanogenum, the effects of various ktzT homologous genes on the biosynthetic efficacy of L‐Piz were further investigated. Five other piperazate synthase gene variants were codon optimized for A. melanogenum (Table S2 and S3), including htmC_Am_ that was derived from the htmC gene of Streptomyces himastatinicus ATCC 53653 (Ma *et al*., 2011), sfaC_Am_ that was derived from sfaC of S. flaveolus DSM 9954 (Qu *et al*., 2011), padO_Am_ that was derived from padO of Streptomyces sp. RJA2928 (Du *et al*., 2013), matF_Am_ that was derived from matF of Actinomadura atramentaria DSM 43919 (Leipoldt *et al*., 2017) and XF36_Am_ that was derived from XF36_RS26795 of Pseudonocardia sp. HH130629‐09 (Sit *et al*., 2015). As a primary selection of a more active homolog, these five genes along with ktzT_Am_ were first fused to 6× His tag gene and individually expressed in Escherichia coli BL21 (DE3) to obtain pure protein, so that their in vitro catalytic activities would help recognize the possibly optimal gene element (Liang *et al*., 2019). As a result, these genes were readily expressed, and the soluble faction was purified using nickel‐affinity chromatography (Fig. 2C). The determination of specific enzyme activities demonstrated that MatF_Am_ and XF36_Am_ exhibited low activities (1.20 ± 0.02 and 0.07 ± 0.08 U mg^‐1^, respectively) among those purified proteins, whereas the remaining four enzymes had higher activities (around 1.60 U mg^‐1^) than these two (Fig. 2D and Fig. S7). Thus, the in vitro catalysing assay could not recognize the optimal gene element of piperazate synthase. To further identify the optimal element among these six genes, the expression cassettes of the latter five genes were constructed and introduced into the DF1 strain (Fig. 1B, Module 2), generating DFK2, DFK3, DFK4 and DFK5 strains, respectively. Then, together with the DFK1 strain, they were, respectively, cultivated in 50 ml of MSP medium in 250‐ml shaking flask for 120 h; HPLC quantification of L‐Piz was then performed. We found that DFK1 could produce the highest titre (179 ± 11 mg l^‐1^) of extracellular L‐Piz as compared with all the other strains (Table S1). Correspondingly, the relative intracellular content of N^5^‐hydroxy‐L‐ornithine, the direct precursor of L‐Piz, exceeded the contents of the remaining five strains harbouring different piperazate genes (*P* < 0.05) (Fig. S2). These results suggested that KtzT_Am_ might have the strongest in vivo catalysing activity in *A*. *melanogenum*.

Furthermore, it was determined that the L‐ornithine synthetic genes (*argA*, *argD* and *argE*) exhibited significant upregulation in the DFK1 strain than in the DFK3, DFK4 and DFK5 strains (Fig. 2A); the transcriptional levels of the L‐ornithine synthetic genes between the DFK1 and DFK2 strains were similar, except that the *argE* from the DFK1 strain was upregulated as compared to that from the DFK2 strain (Fig. 2A). These reasons might have contributed to the highest intracellular L‐ornithine content in the DFK1 strain, although it was only slightly higher than that in the DFK2 strain (Table S1). These superior metabolic intensity and metabolite accumulation in the DFK1 strain, compared with those in the other strains, could be attributed to the strong in vivo activity of KtzT_Am_ that pulled more carbon fluxes to flow into the L‐Piz synthetic cascade (d'Espaux *et al*., 2017; Lu *et al*., 2019a,2019b) starting from L‐ornithine biosynthesis (Fig. 1). Based on all these results, we proposed that *ktzT_Am_* was the optimal piperazate synthase gene element that most adapted to *A*. *melanogenum*, which might be due to the fact that the translated protein KtzT_Am_ could be better post‐modified in this yeast than in the other ones.

### Enhanced extracellular titre of l‐Piz

Following the development of the strain DFK1, a higher production of L‐Piz was pursed to pave the basis for its possible scale‐up production as a fine chemical. It should be noted that MSP medium [2.5% sucrose, 0.4% (NH_4_)_2_SO_4_, 0.3% K_2_HPO_4_, 0.075% citric acid, 0.008% MgSO_4_, 0.0002% ZnSO_4_ and 0.17% yeast nitrogen base (YNB) without amino acids, (w/v)] (Lu *et al*., 2019a,2019b) was used to establish iron‐depleted conditions. As described above, the *sidA* gene has not been modified; thus, its transcription would be strongly inhibited under the presence of iron in the medium (Chi *et al*., 2013). This iron‐activated transcriptional repression is driven by the GATA‐type repressor SreA, as demonstrated in previous studies (Chi *et al*., 2013; Lu *et al*., 2019a,2019b). Therefore, the iron‐replete conditions established using the MSP medium could secure the expression of *sidA* intracellularly, such as to catalyse the conversion of L‐ornithine into N^5^‐hydroxy‐L‐ornithine.

However, there were some shortcomings regarding the MSP medium that made it non‐ideal for the production of L‐Piz: (i) its carbon source was sucrose, which can be more expensive than glucose; (ii) the supplementation of extra L‐arginine in the medium was indispensable for maintaining the propagation of the L‐arginine auxotrophic DFK1 strain, as it was for the original strain of DOLC19 (Lu *et al*., 2019a,2019b); (iii) the use of this medium led to low cell density of the DFK1 strain (approximately 6 g L^‐1^; Table S1), as was noted for the original strain of DOLC19 (Lu *et al*., 2019a,2019b); and (iv) the use of the medium led to low transcriptional levels of the *sidA* and *ktzT_Am_* genes (Fig. 2B), which was the most likely reason for the low titre of L‐Piz produced by this strain (Table S1). To tackle these issues, the use of iron‐replete complete media, such as the yeast extract peptone dextrose (YPD) medium, might be effective. However, another problem emerges in this situation: how to enable the robust iron regulation‐free transcription of the *sidA* gene under iron‐replete conditions? The replacement of its native promoter with a constitutively initiating one could be a feasible solution.

Therefore, the native *sidA* gene in the DF1 strain was first deleted, generating the DFA1 mutant strain. Then, the genetic cascade of *sidA‐ktzT_Am_*, with each gene under the control of a constitutive promoter, was introduced into the DFA1 strain (Fig. 1B, Module 3); the reconstituted strain was then designated DFAK1. As shown in Fig. 2B, the transcriptional level of *sidA* in the DFAK1 strain was greatly elevated, by approximately 19‐fold, in an iron‐replete complete medium (CM) [3.73% glucose, 1.4% peptone, 0.3% K_2_HPO_4_, 0.075% citrate monohydrate, 0.008% MgSO_4_, 0.0002% ZnSO_4_ and 0.32% YNB, (w/v)] as compared with that in the DFK1 strain, which clearly indicated that the de‐repression of iron on *sidA* was achieved under iron‐replete conditions. In addition, using CM, the *ktzT_Am_* gene in the DFAK1 strain was upregulated by approximately onefold as compared with that in the DFK1 strain (Fig. 2B). Benefiting from this transcriptional de‐repression of iron for the *sidA* gene and the upregulation of *ktzT_Am_* gene, the DFAK1 strain was capable of producing 695 ± 41 mg l^‐1^ L‐Piz with 9.85 ± 0.21 g l^–1^ of dry cell weight after 120 h of cultivation in 50 ml CM in 250‐ml shaking flasks, without requiring extra L‐arginine (Table S1). These results demonstrate that by constructing the *A*. *melanogenum* DFAK1 strain (Fig. 1B), the fermentation production of L‐Piz has been improved by the use of a cheaper and more convenient medium, yielding a higher titre. Thus, an increased L‐Piz titre could be expected when the production is scaled up in bioreactors.

In addition, we determined that the intracellular L‐ornithine content for the DFAK1 strain cultivated in the CM medium increased by approximately twofold as compared to that for the rest of the engineered strains in MSP medium at 120 h (Table S1). This result can be ascribed to the robust metabolism of L‐Piz synthesis generated from the reconstituted *sidA‐ktzT_Am_* cascade, which pulled up the L‐ornithine biosynthesis. More importantly, this phenomenon indicates that the DFAK1 strain has the potential to further produce more L‐Piz from the L‐ornithine that is excessively available with more subsequent genetic modifications.

### L‐Piz production with a 10‐L batch and fed‐batch fermentation

**Fig. 3:**
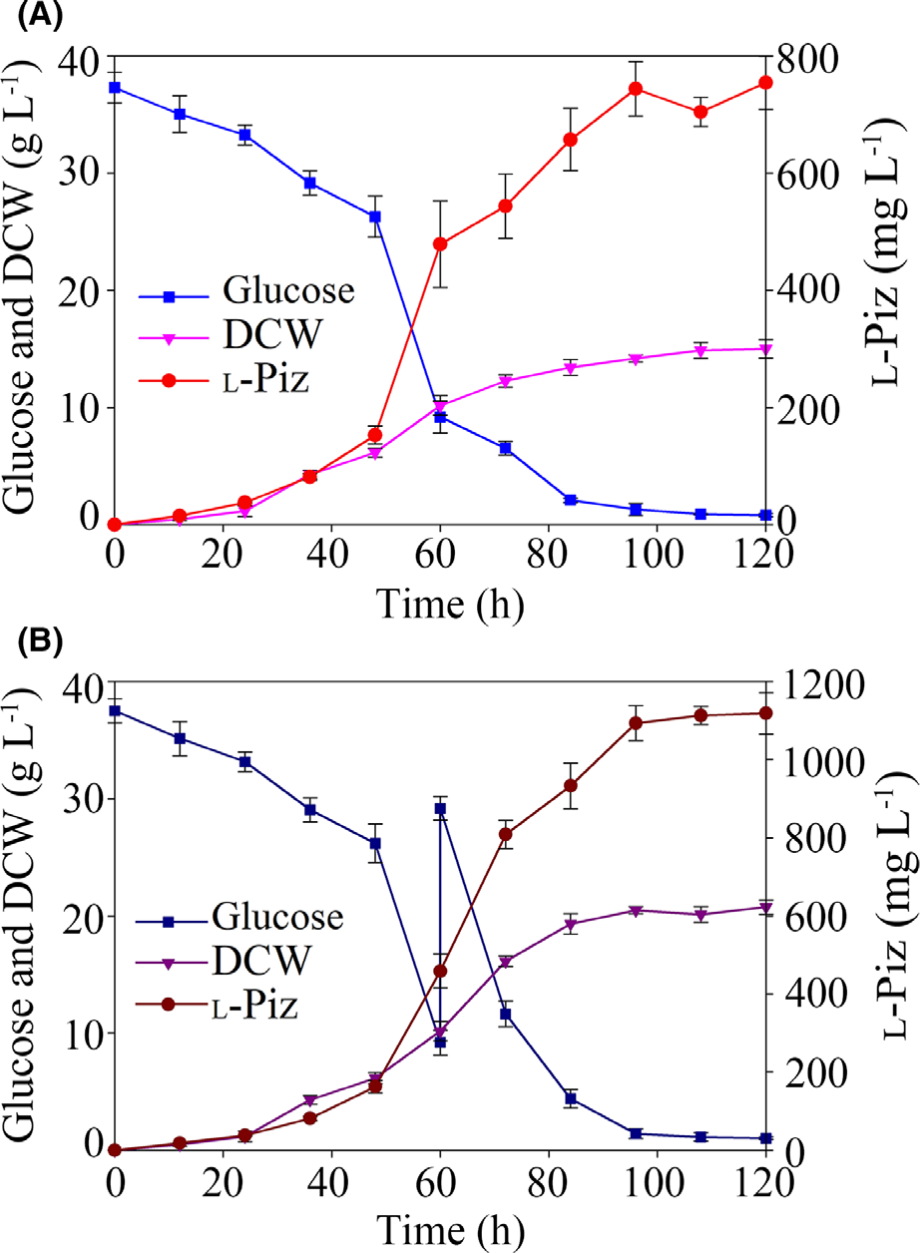
Monitoring of the extracellular titre of L‐Piz, dry cell weight and residual glucose during the 120‐h 10‐l batch fermentation (A) and 10‐l fed‐batch fermentation (B) with the *A*.*melanogenum* DFAK1 strain. Data are presented as mean ± SD; *n* = 3.

Subsequent to the flask fermentation, a 10‐L fermentation of L‐Piz was implemented using the DFAK1 strain in CM. In the batch fermentation, an extracellular L‐Piz titre of 754 ± 45 mg l^‐1^ and a dry cell weight of 15.02 ± 0.79 g l^‐1^ were reached after 120 h of fermentation (Fig. 3A). Meanwhile, the residual glucose at the end of this fermentation was as low as 0.80 ± 0.13 g l^‐1^ (Fig. 3A). These data demonstrate that the initial glucose was almost fully utilized to support a L‐Piz yield of 21 ± 1 mg g^‐1^ glucose as well as a more robust cell growth, contributing to the higher L‐Piz titre. It was noted that at 60 h of the batch fermentation, the cells were about to reach the stationary phase, which was accompanied with a significant drop in the residual glucose (Fig. 3A). To further increase production, a fed‐batch fermentation with feeding of glucose at 60 h was designed. As shown in Fig. 3B, with 120.0 g glucose supplemented to the 10‐l bioreactor at the 60th hour of the L‐Piz fermentation, a final titre of 1.12 ± 0.05 g l^‐1^ for L‐Piz could be achieved. At the end of the fed‐batch fermentation, the residual glucose was below 0.8 g l^‐1^ and the cell mass exceeded 20 g l^‐1^ of dry weight, demonstrating the enhanced conversion of glucose to biomass and a high L‐Piz yield (21 ± 1 mg g^‐1^ glucose), as the same as that obtained in the batch fermentation (*P* < 0.05). To the best of our knowledge, this study is the first report on the gram‐scale production of L‐Piz from glucose at high yield in a cell factory. This bench‐scale fermentation underlined the feasibility for the future scale‐up production of L‐Piz in pilot or even industrial plants under proper conditions that need to be investigated and optimized.

In conclusion, L‐Piz was successfully synthesized using a completely biological process in *A*. *melanogenum* by reconstituting a robust genetic cascade of native L‐ornithine‐N^5^‐hydroxylase tandem the optimal piperazate synthase within, achieving a gram‐scale extracellular titre. This fungal cell factory can produce L‐Piz via one‐step direct conversion from feedstock by fermentation, without depending on any chemically synthesized precursor or harsh conditions involving the utilization of extreme temperatures and organic solvents; this method thus shows advantages over the conventional chemical synthesis of L‐Piz with regard to green chemistry. This approach is promising as an alternative to the current chemical synthesis of L‐Piz.

## Conflict of interest

The authors declare no conflicts of interest to this work. The strains in this work can only be shared upon research collaboration or technology transfer due to patent protection.

## Supporting information


**Fig. S1.** (A) High‐performance liquid chromatography (HPLC) analysis of extracellular siderophore components of fusarinine C (FsC) and fusarinine B (FsB) produced by the genetically modified *A*. *melanogenum* DOLC19 strain; (B) HPLC analysis of extracellular siderophore components produced by the *A*. *melanogenum* DF1 strain; (C) HPLC analysis of the Fmoc‐Cl‐derivatized extracellular product from the engineered *A*. *melanogenum* strains of this study against that of the L/D‐piperazic acid standard; (D) absolute configuration of extracellular product from the engineered *A*. *melanogenum* strain DFK1.
**Fig. S2.** Quantification of N^5^‐hydroxy‐L‐ornithine intermediate in each strain harboring different piperazate synthase encoding genes against that of their starting strain *A*. *melanogenum* DOLC19. Data are presented as mean ± standard deviation; *n* = 3.
**Fig. S3.** Extracted ion chromatography the of Fmoc‐Cl derivatized Piz standard (A) and extracellular fungal product (C) and corresponding LC‐MS spectrums analysis thereof (B, D). Mass‐to‐charge ratios (*m/z*) of 353.19 ([M + H]^+^) and 705.14 ([2 M+H]^+^) (highlighted in red diamonds) corresponded closely to that of the Fmoc‐Piz derivatives (353.18 and 705.12).
**Fig. S4.** Elution chromatography of Fmoc‐Cl derivatized extracellular fungal product from preparative HPLC, the purposed eluted portion was boxed with two dotted‐line (A); HPLC analysis of the resulting elution from preparative HPLC (B) as compared with the HPLC analysis of Fmoc‐Cl derivatized Piz standard (C).
**Fig. S5.** ^1^H‐NMR (A) and ^13^C‐NMR (B) spectra and analyses for the Fmoc‐removed product of suspicious Fmoc‐Piz. The NMR data were calibrated according to the standard chemical shift of DMSO‐d6. ^1^H‐NMR: (500 MHz, DMSO‐d6) δ 2.89 (s, 1H), 2.14 (s, 1H), 1.61‐1.89 (m, 1H), 1.16‐1.23 (m, 1H); ^13^C NMR: (125 MHz, DMSO‐d6) δ 1171.68, 58.12, 43.93, 25.70, 22.02.
**Fig. S6.** Extracted total ion chromatography of L‐FDAA derivatized Piz standard (A) and the extracellular fungal product (C) and the corresponding LC‐MS spectrums thereof (B, D). Mass‐to‐charge ratios (*m/z*) of 383.09 ([M + H]^+^) (highlighted red diamonds) was in consistence to that of the L‐FDAA‐Piz derivatives (383.09).
**Fig. S7.** HPLC quantification of L‐Piz produced by the in vitro catalysis of purified piperazate synthases with the N^5^‐hydroxy‐L‐ornithine as the substrate. The peak area for the sample of SfaC, KtzT, PadO, MatF, HmtC and XF36, corresponded to a final L‐Piz content of 549 ± 8, 545 ± 30, 506 ± 24, 491 ± 17, 340 ± 6, 237 ± 2 mg mL^‐1^, respectively.
**Fig. S8.** Calibration curves of L‐piperazic acid (A) and N^5^‐hydroxy‐L‐ornithine (B) against their peak areas from HPLC analysis.
**Table S1.** Quantification of the intracellular L‐ornithine content and extracellular titer of L‐piperazic acid and siderophores for all the *A*. *melanogenum* strains in this study.
**Table S2.** Codon optimized sequences of piperazate synthase genes for *A*. *melanogenum* used in this study.
**Table S3.** Piperazate synthase genes from different hosts and their Genbank accession numbers
**Table S4.** DNA sequence for the promoter of phosphoglycerate kinase gene amplified from genomic DNA of *A*. *melanogenum* HN6.2 strain.
