# Supplementary material for: Bioproduction of L‐piperazic acid in gram scale using Aureobasidium melanogenum
Source: Microb Biotechnol. 2021 Jun 3;14(4):1722–9. doi: 10.1111/1751-7915.13838 (PMC8313269; doi:10.1111/1751-7915.13838)
Supplement: Supplementary file 1 — Fig. S1. (A) High‐performance liquid chromatography (HPLC) analysis of extracellular siderophore components of fusarinine C (FsC) and fusarinine B (FsB) produced by the genetically modified A. melanogenum DOLC19 strain; (B) HPLC analysis of extracellular siderophore components produced by the A. melanogenum DF1 strain; (C) HPLC analysis of the Fmoc‐Cl‐derivatized extracellular product from the engineered A. melanogenum strains of this study against that of the L/D‐piperazic acid standard; (D) absolute configuration of extracellular product from the engineered A. melanogenum strain DFK1. Fig. S2. Quantification of N5‐hydroxy‐L‐ornithine intermediate in each strain harboring different piperazate synthase encoding genes against that of their starting strain A. melanogenum DOLC19. Data are presented as mean ± standard deviation; n = 3. Fig. S3. Extracted ion chromatography the of Fmoc‐Cl derivatized Piz standard (A) and extracellular fungal product (C) and corresponding LC‐MS spectrums analysis thereof (B, D). Mass‐to‐charge ratios (m/z) of 353.19 ([M + H]+) and 705.14 ([2 M+H]+) (highlighted in red diamonds) corresponded closely to that of the Fmoc‐Piz derivatives (353.18 and 705.12). Fig. S4. Elution chromatography of Fmoc‐Cl derivatized extracellular fungal product from preparative HPLC, the purposed eluted portion was boxed with two dotted‐line (A); HPLC analysis of the resulting elution from preparative HPLC (B) as compared with the HPLC analysis of Fmoc‐Cl derivatized Piz standard (C). Fig. S5. 1H‐NMR (A) and 13C‐NMR (B) spectra and analyses for the Fmoc‐removed product of suspicious Fmoc‐Piz. The NMR data were calibrated according to the standard chemical shift of DMSO‐d6. 1H‐NMR: (500 MHz, DMSO‐d6) δ 2.89 (s, 1H), 2.14 (s, 1H), 1.61‐1.89 (m, 1H), 1.16‐1.23 (m, 1H); 13C NMR: (125 MHz, DMSO‐d6) δ 1171.68, 58.12, 43.93, 25.70, 22.02. Fig. S6. Extracted total ion chromatography of L‐FDAA derivatized Piz standard (A) and the extracellular fungal product (C) and the c [file MBT2-14-1722-s001.docx]

**Supporting Information**

**Bioproduction of L-piperazic acid in gram scale using *Aureobasidium melanogenum***

Cuncui Kong^1^, Zhuangzhuang Wang^1^, Guanglei Liu^1, 2^, Zhenming Chi^1, 2^, Rodrigo Ledesma-Amaro^3^, Zhe Chi^1, 2,^ *

^1^College of Marine Life Sciences, Ocean University of China, No.5 Yushan Road, 266003 Qingdao, China.

^2^Pilot National Laboratory for Marine Science and Technology, No.1 Wenhai Road, 266237 Qingdao, China.

^3^Department of Bioengineering, Imperial College London, London SW7 2AZ, UK.

* Corresponding Author:

Zhe Chi

Email: cz1108@ouc.edu.cn

Tel: +86-15953284211

Fax: +86-532-82032266


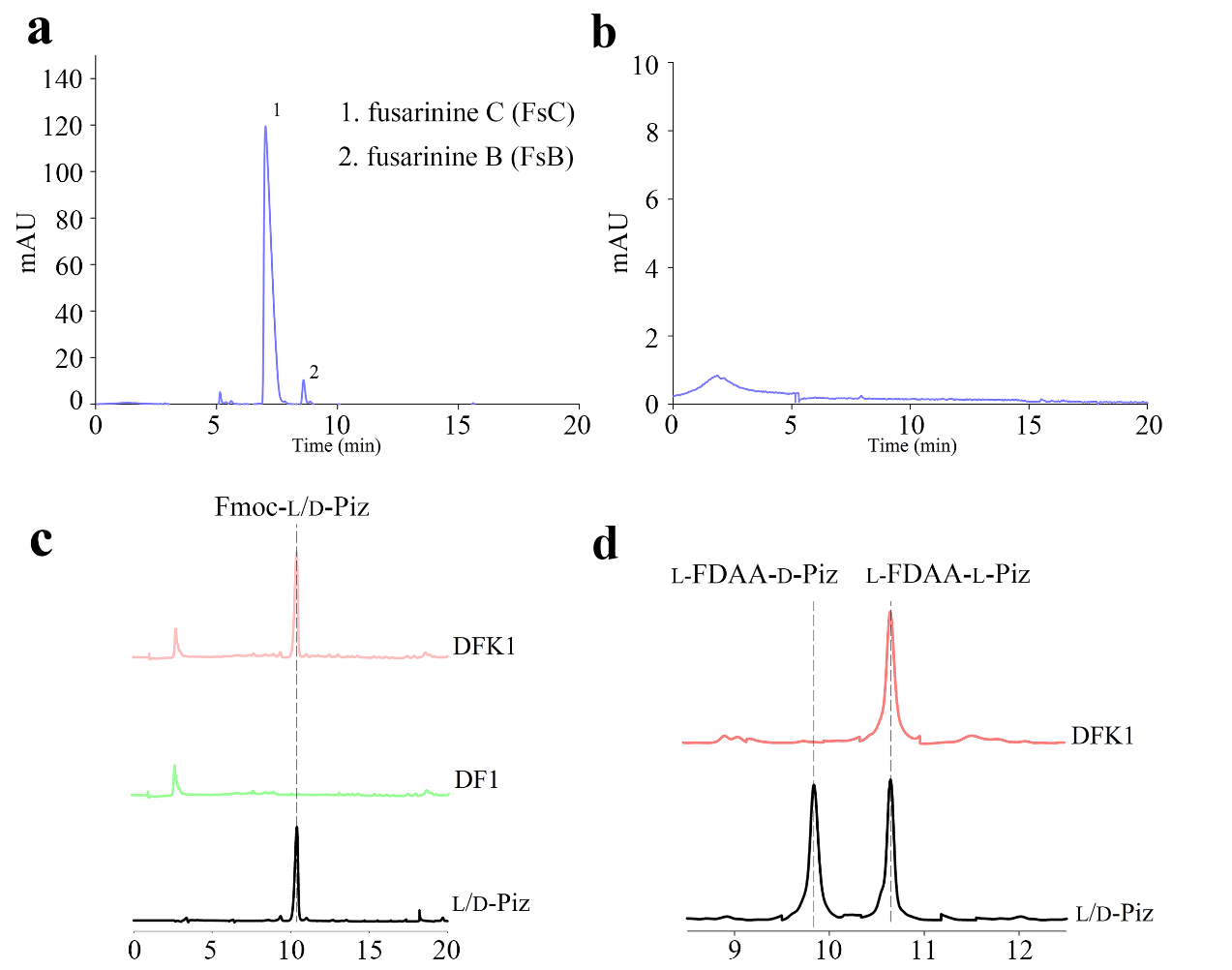


**Figure S1.** (a) High-performance liquid chromatography (HPLC) analysis of extracellular siderophore components of fusarinine C (FsC) and fusarinine B (FsB) produced by the genetically modified *A. melanogenum* DOLC19 strain; (b) HPLC analysis of extracellular siderophore components produced by the *A. melanogenum* DF1 strain; (c) HPLC analysis of the Fmoc-Cl-derivatized extracellular product from the engineered *A. melanogenum* strains of this study against that of the L/D-piperazic acid standard; (d) absolute configuration of extracellular product from the engineered *A. melanogenum* strain DFK1.


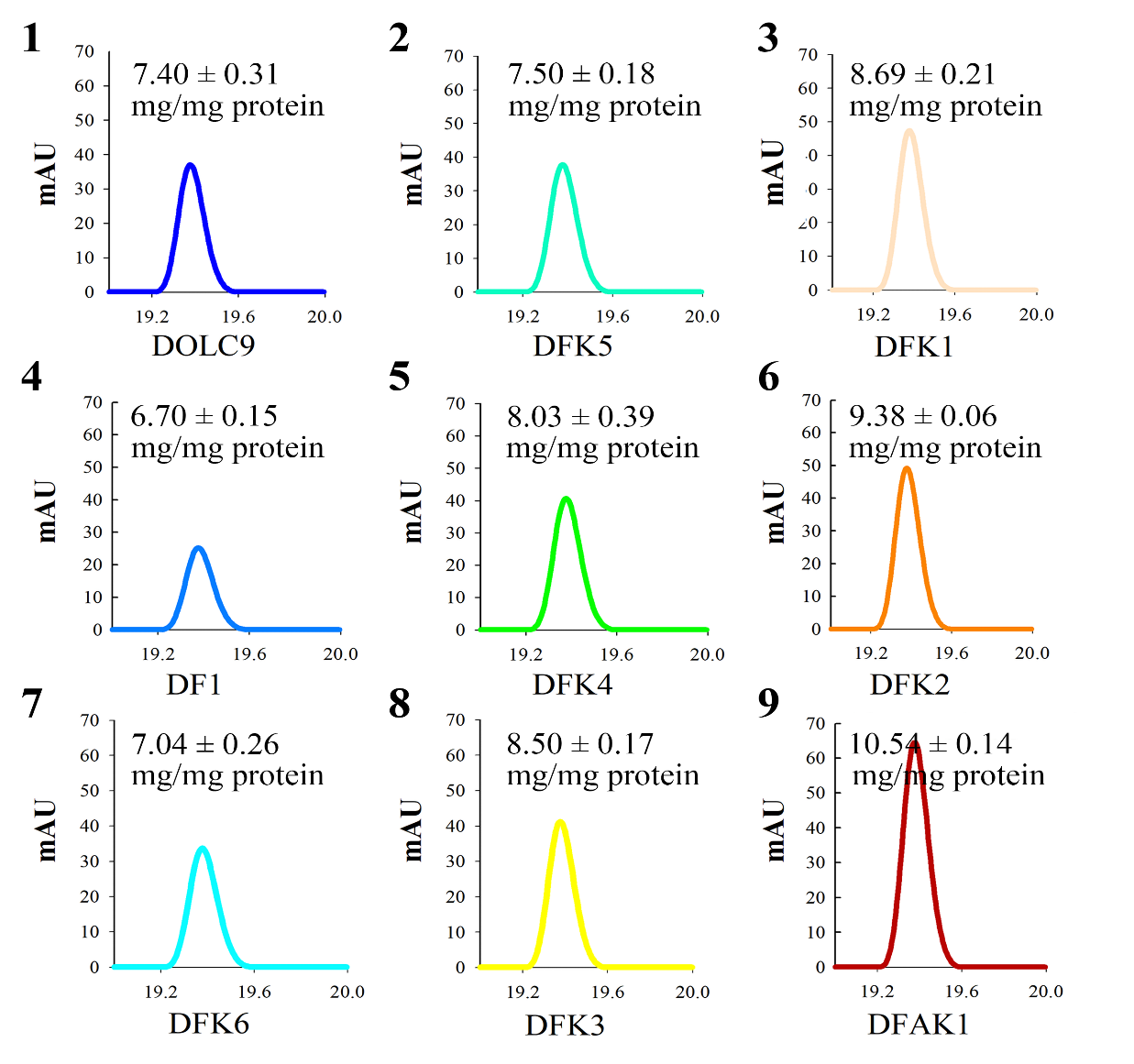


**Figure S2.** Quantification of N^5^-hydroxy-L-ornithine intermediate in each strain harboring different piperazate synthase encoding genes against that of their starting strain *A. melanogenum* DOLC19. Data are presented as mean ± standard deviation; n = 3.


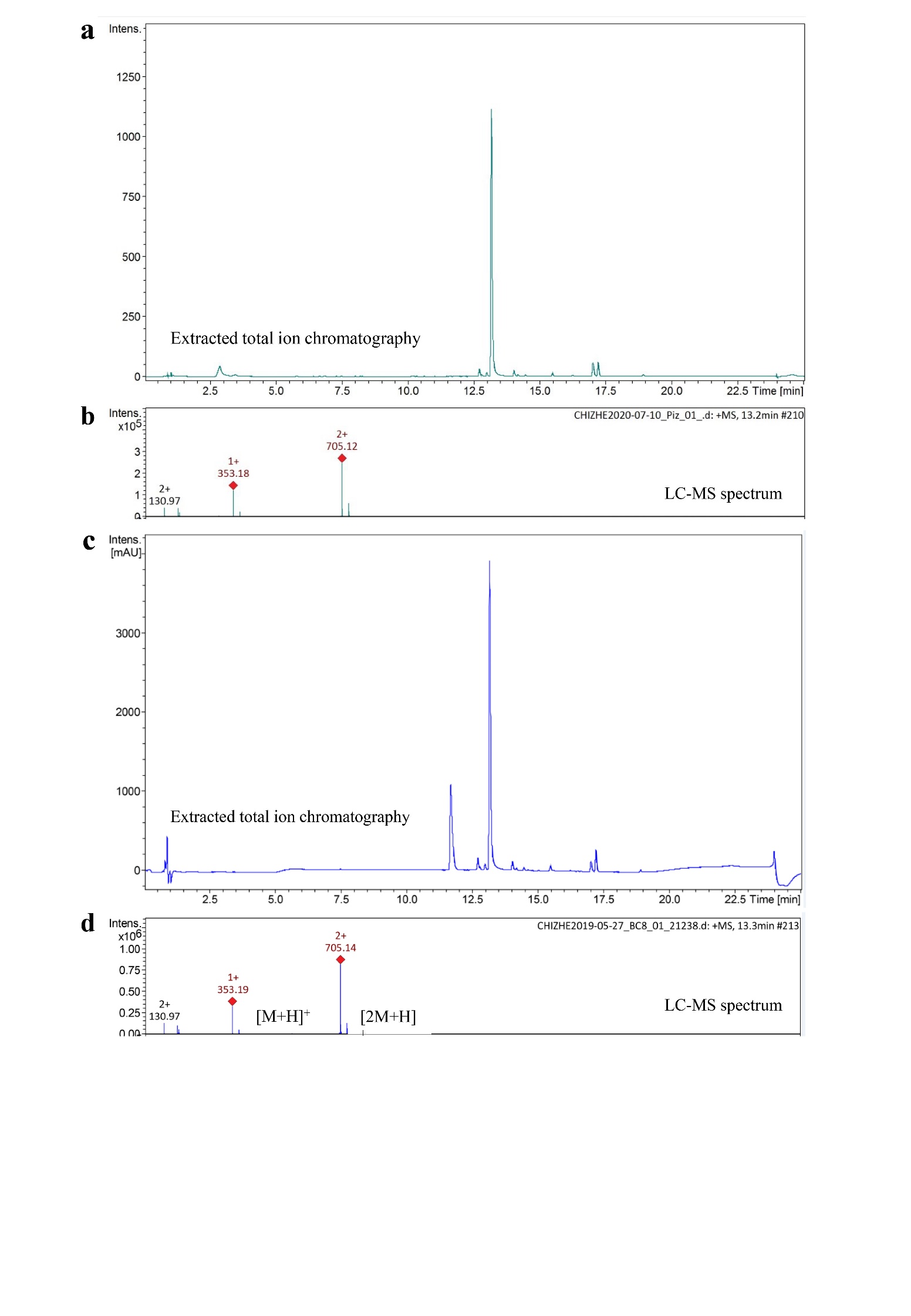


LC-MS spectrum

**Figure S3.** Extracted ion chromatography of Fmoc-Cl derivatized Piz standard (a) and extracellular fungal product (c) and the corresponding LC-MS spectrums analysis thereof (b, d). Mass-to-charge ratios (*m/z*) of 353.19 ([M+H]^+^) and 705.14 ([2M+H]^+^) (highlighted in red diamonds) corresponded closely to that of the Fmoc-Piz derivatives (353.18 and 705.12).


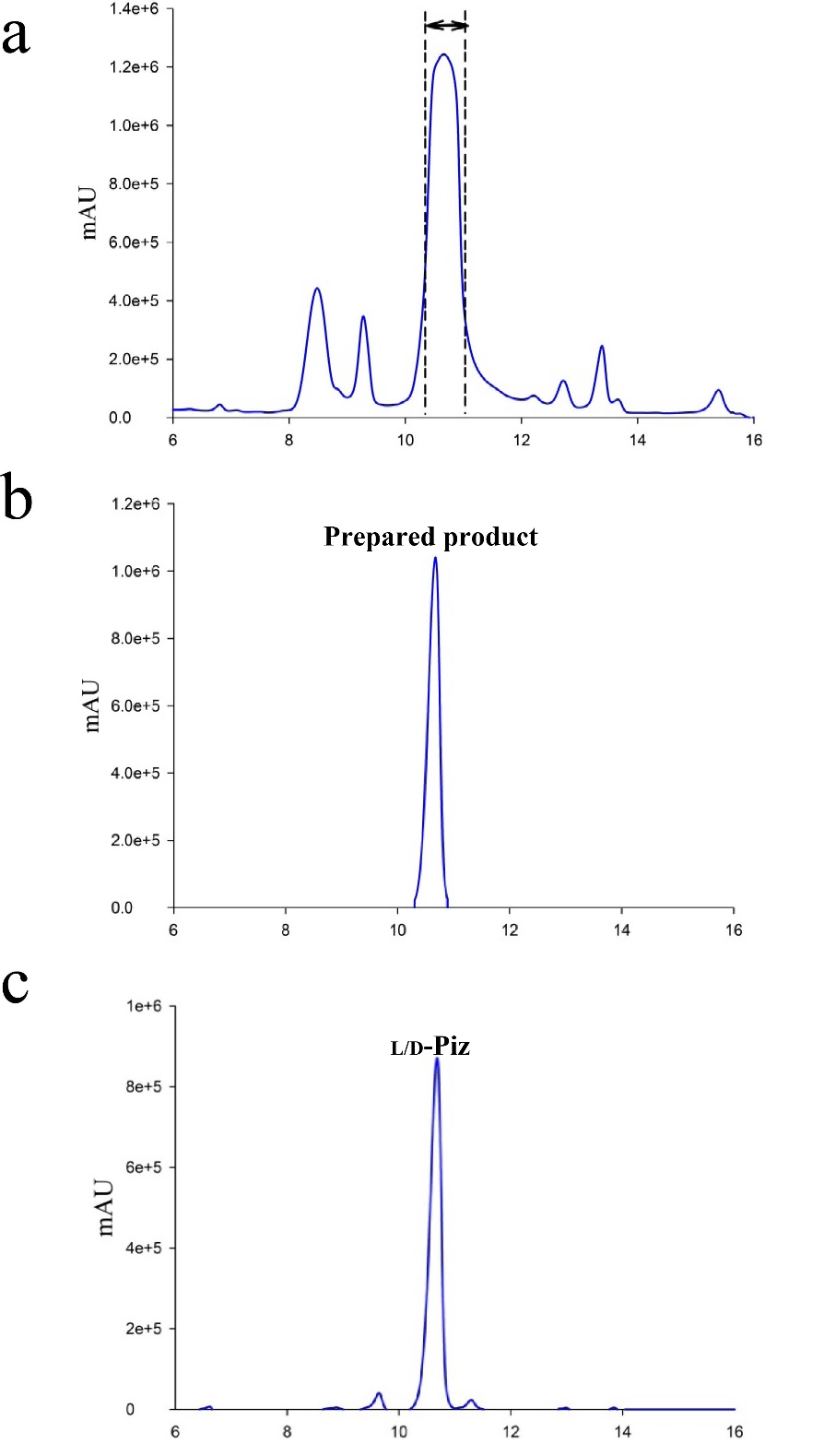


**Figure S4.** Elution chromatography of Fmoc-Cl derivatized extracellular fungal product from preparative HPLC, the purposed eluted portion was boxed with two dotted-line (a); HPLC analysis of the resulting elution from preparative HPLC (b) as compared with the HPLC analysis of Fmoc-Cl derivatized Piz standard (c).


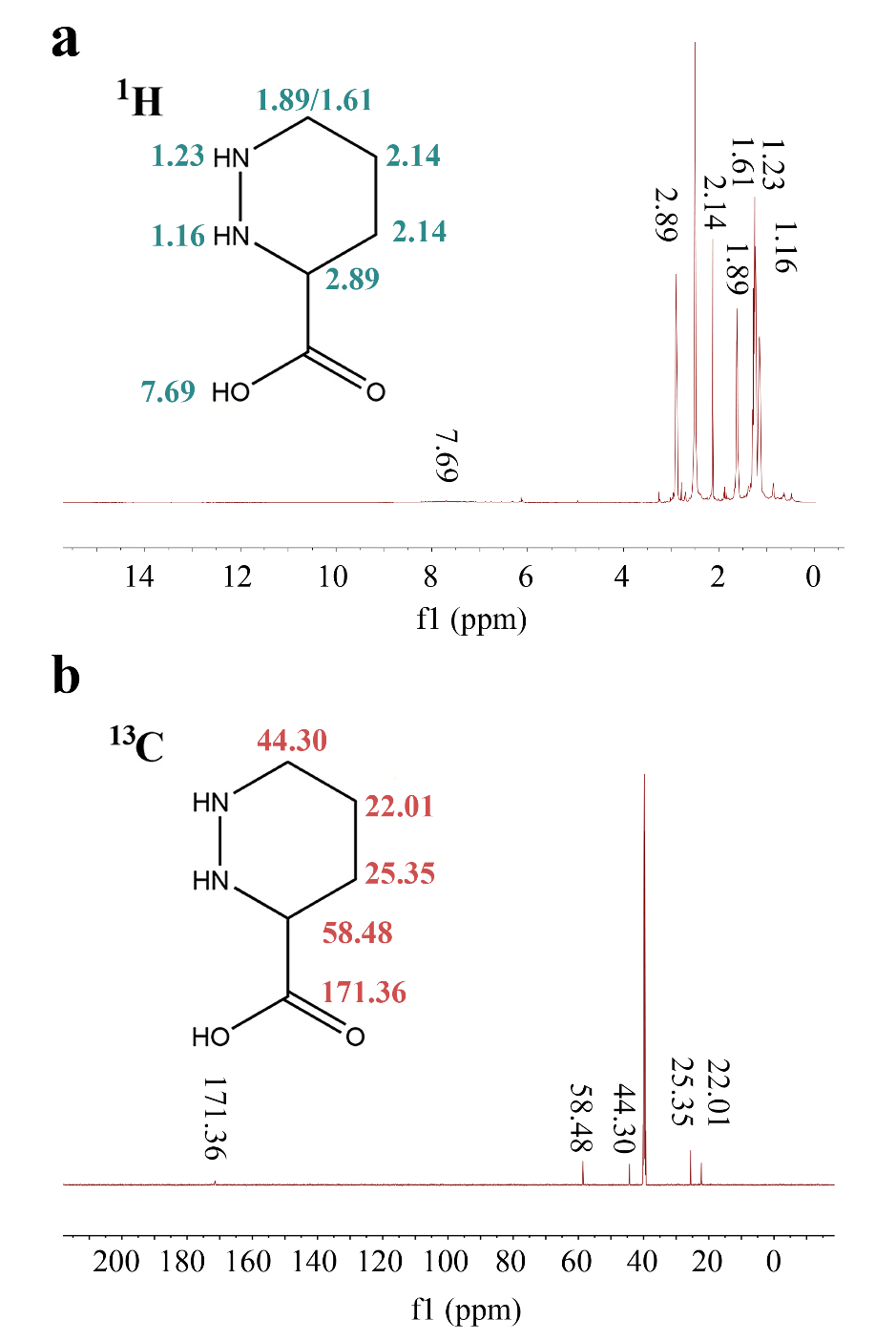


**Figure S5.** ^1^H-NMR (a) and ^13^C-NMR (b) spectra and analyses for the Fmoc-removed product of suspicious Fmoc-Piz. The NMR data were calibrated according to the standard chemical shift of DMSO-d6. ^1^H-NMR: (500 MHz, DMSO-d6) δ 2.89 (s, 1H), 2.14 (s, 1H), 1.61-1.89 (m, 1H), 1.16-1.23 (m, 1H); ^13^C NMR: (125 MHz, DMSO-d6) δ 1171.68, 58.12, 43.93, 25.70, 22.02.

In the ^1^H-NMR spectrum (Fig. S5a), the multiple peaks with high field signals of 1.61–1.89 ppm were the hydrogen characteristic signals of methyl or methylene, later confirmed as methylene hydrogen signals via the integration of signal peaks. The peak with a high field signal of 2.89 ppm was confirmed to be the methylidyne signal via the integration of signal peaks. The hydrogen signals of methylene in pairs were found at a chemical shift of 2.14 ppm. The multiple peaks of high field signals 1.16–1.23 ppm might have been the characteristic peak signals of cycloiminohydrogen, with a high density of electron cloud and strong shielding effect. The peak in the low filed of 7.69 ppm might have been ascribed to the hydrogen of the carboxyl group; this weak signal could be attributable to the over-activity of carboxyl in solution. In the ^13^C-NMR spectrum (Fig. S5b), the signal peak of 171.68 in the low field was the characteristic signal of the carbonyl carbon. Thus, the hydroxyl hydrogen with a chemical shift of 7.69 ppm in the hydrogen spectrum was confirmed to belong to the carboxyl group. The characteristic peaks of hydrocarbon carbon were 58.12, 43.93, 25.70, and 22.02 ppm in the high field. The carbon atoms with chemical shifts of 58.46 was methylidyne, and the carbon atoms with chemical shifts of 43.93, 25.35 and 22.02 ppm were methylene. The NMR of skeleton structure acid in our work closely matched with the reported skeleton structure of hexahydropyridazin-3-ones (Chagarovskiy*, et al.*, 2017). Based on the analysis of the NMR data, the compound was verified as Piz.


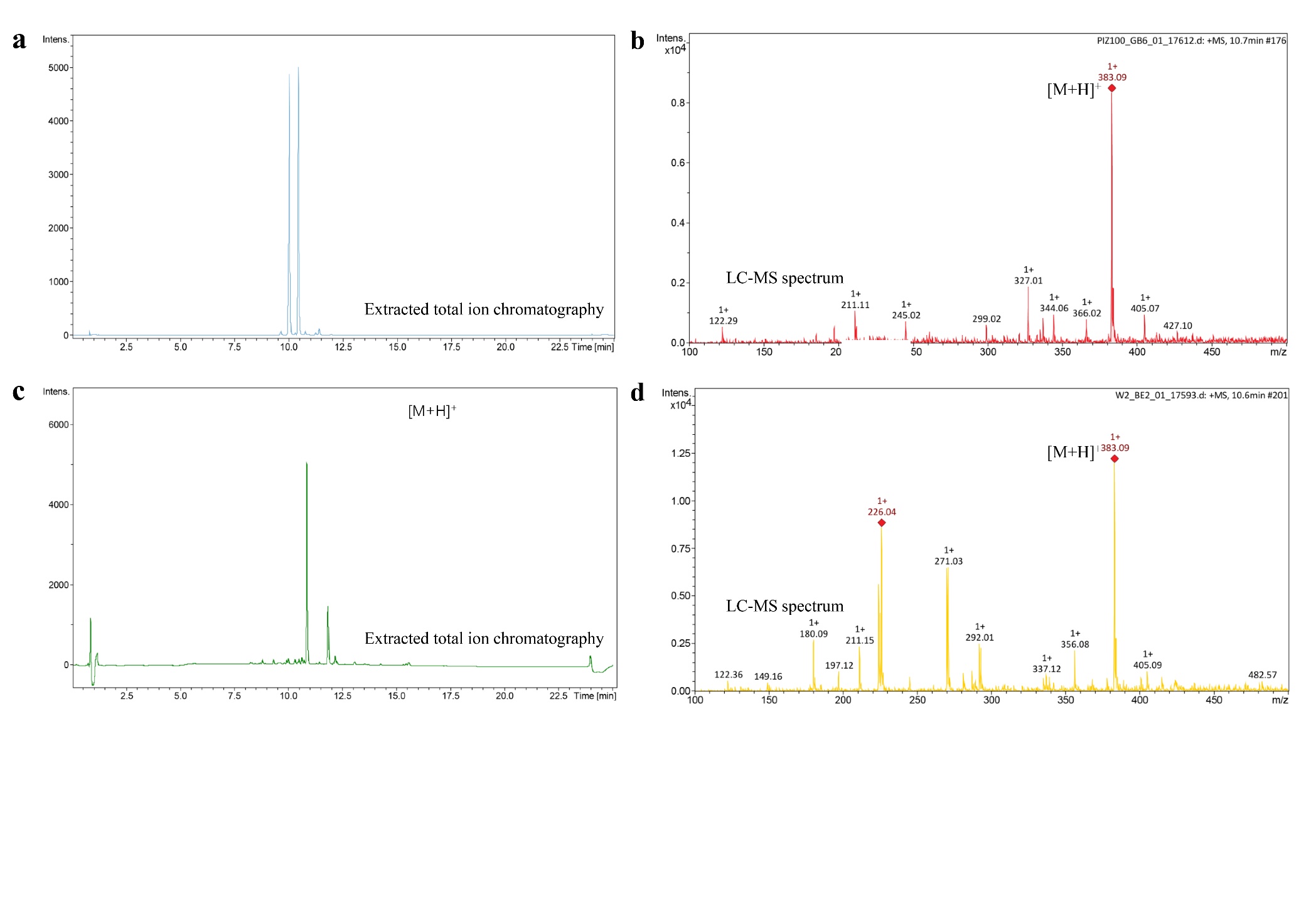


**Figure S6.** Extracted total ion chromatography of L-FDAA derivatized Piz standard (a) and the extracellular fungal product (c) and the corresponding LC-MS spectrums thereof (b, d). Mass-to-charge ratios (*m/z*) of 383.09 ([M+H]^+^) (highlighted red diamonds) was in consistence to that of the L-FDAA-Piz derivatives (383.09).


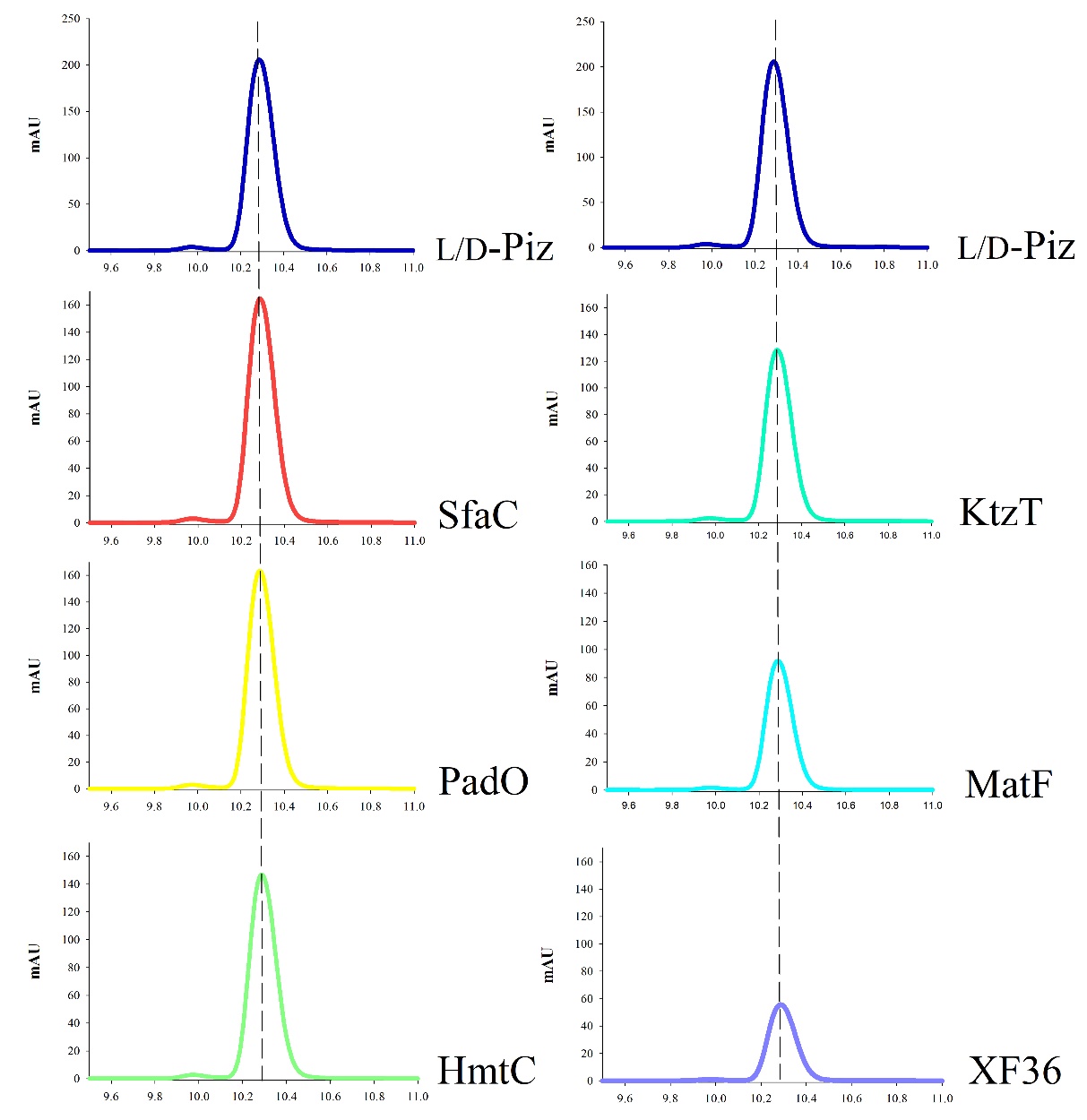


**Figure S7.** HPLC quantification of L-Piz produced by the in vitro catalysis of purified piperazate synthases with the N^5^-hydroxy-L-ornithine as the substrate. The peak area for the sample of SfaC, KtzT, PadO, MatF, HmtC and XF36, corresponded to a final L-Piz content of 549 ± 8, 545 ± 30, 506 ± 24, 491 ± 17, 340 ± 6, 237 ± 2 mg mL^-1^, respectively.


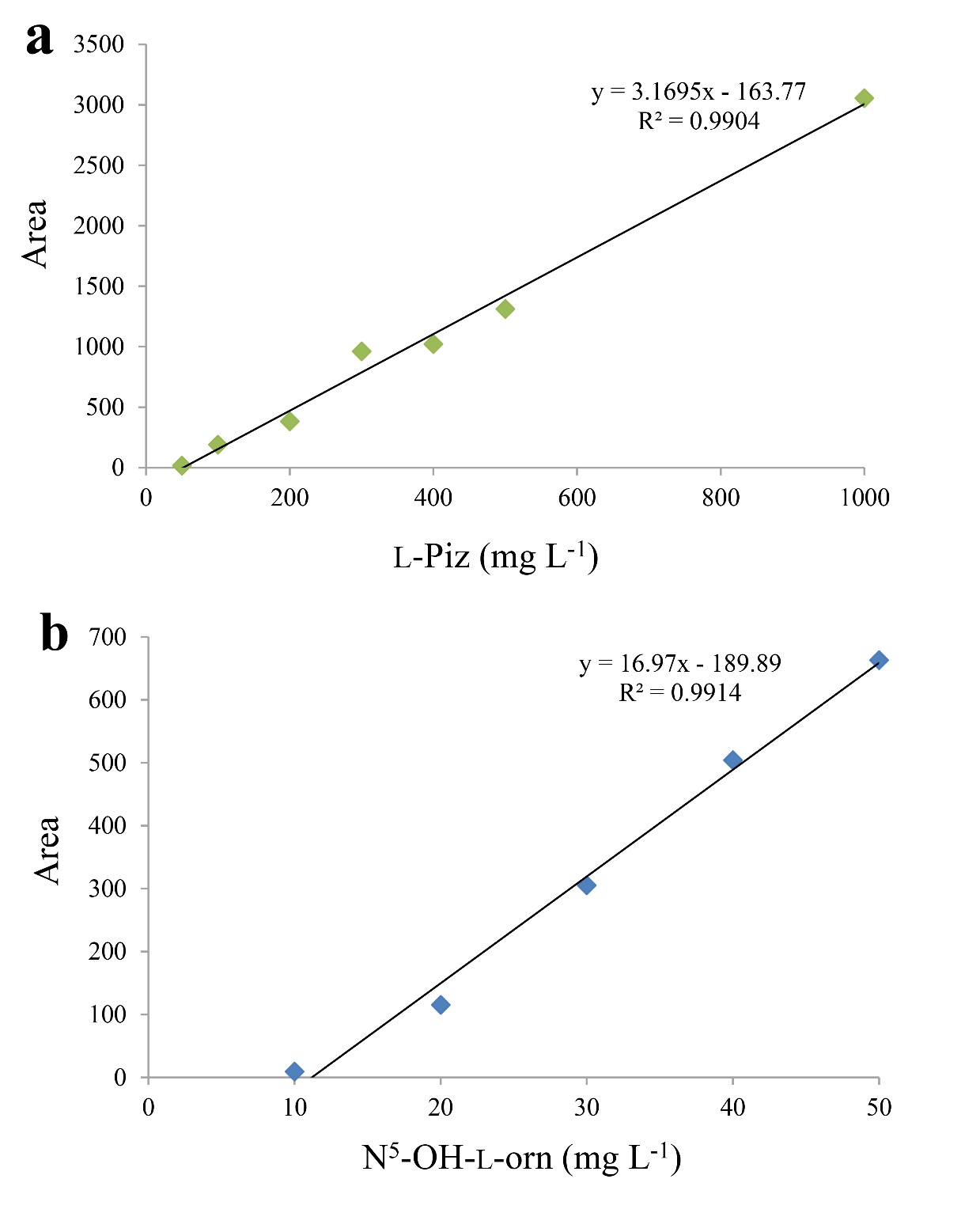


**Figure S8.** Calibration curves of L-piperazic acid (a) and N^5^-hydroxy-L-ornithine (b) against their peak areas from HPLC analysis.

**Table S1.** Quantification of the intracellular L-ornithine content and extracellular titer of L-piperazic acid and siderophores for all the *A. melanogenum* strains in this study.

| Strains | Intracellular L-ornithine [mg (mg protein)^–1^] | Extracellular L-piperazic acid (mg L^–1^) | Extracellular siderophores (mM) | DCW (g) |
| --- | --- | --- | --- | --- |
| DOLC19 (starting strain) | 1.80 ± 0.01^bc^ | / | 1.65 ± 0.05 | 6.43 ± 0.14^b^ |
| DF1 (D*sidF*) | 1.58 ± 0.12^c^ | / | ND | 5.22 ± 0.21^c^ |
| DFK1 (D*sidFPtef:ktzT_Am_*) | 1.98 ± 0.04^b^ | 179 ± 11^b^ | ND | 6.52 ± 0.06^b^ |
| DFK2 (D*sidFPtef:htmC_Am_*) | 1.80 ± 0.01^bc^ | 153 ± 5^c^ | ND | 6.42 ± 0.62^b^ |
| DFK3 (D*sidFPtef:safC_Am_*) | 1.61 ± 0.03^c^ | 63 ± 10^d^ | ND | 5.15 ± 0.03^c^ |
| DFK4 (D*sidFPtef:padO_Am_*) | 1.60 ± 0.21^c^ | 48 ± 11^d^ | ND | 5.32 ± 0.31^c^ |
| DFK5 (D*sidFPtef:matF_Am_*) | 1.60 ± 0.11^c^ | 2.47 ± 0.39^e^ | ND | 5.15 ± 0.22^c^ |
| DFK6 (D*sidFPtef:XF36_Am_*) | 1.57 ± 0.04^c^ | 0.14 ± 0.11^f^ | ND | 5.04 ± 0.17^c^ |
| DFAK1 (D*sidF*D*sidAPtef:sidA_Am_-Ppgk:ktzT_Am_*)* | 3.72 ± 0.09^a^ | 695 ± 41^a^ | ND | 9.85 ± 0.21^a^ |

ND, not detectable; DCW, dry cell weight in 1 L of fermentation broth; *, strain was cultivated in iron-replete CM, whereas other strains were cultivated in iron-depleted MSP medium; data are presented as mean ± standard deviation, n = 3; ^abcdef^, data with different superscripts in same column had significant differences.

**Table S2.** Codon optimized sequences of piperazate synthase genes for *A. melanogenum* used in this study.

| Genes | Genbank accession No. | Optimized sequences |
| --- | --- | --- |
| *ktzT_Am_* | MT881544 | ATGTTCGTCCCTGGCCCATACCACGCTCCTGAGGATCGCTGGCTCGTCGATCTTGTCCGTGGTCACCCACTGGCTCAGCTCGCTTCTAACGGTGCTGGTGGTGCTGCTCCACACATCACCCACGTCCCAATCATCGTCGATCCCGAGCTTGATGGTCCTGTCGATCGCCTCGTCGGCATCACTCTTTGGGGTCACATGAACCGTGCTAACCCTCACTGGGCTGCTCTTGGTGGTGCTGCTAACGTCGTCGCTACCTTCGCCGGCCCAAACGCTTACGTCTCTCCCGCCGTCTACAGAACTGCTCCCGCTGCCCCTACCTGGAACTTCACTTCTGTCCAGGTCCGTGGCGAGCTTAGAAAGGTCGAGTCTGCCGACGATACCCTGGCTACTGTCCGTGCCACCGTCGCTGCCCTCGAGTCTAGATTCGGCGCTGGTTGGGATATGACTGGCTCTCTGGACTACTTCCGTCGTATCCTCCCAGGTGTCGGTGCCTTCAGACTTCGCGTCGCTGAGGCCGATGGCATGTTCAAGCTGTCTCAAGAGCAGCAACCTGCCATCAGACGCCGTGTCCGCCACTCTTTCGGCGGTTGCGAGGCTACCAGAGCTGTCGCCGGCCTTATGGACCGCCTGCCTACTGAGCACCACCACCACCACCACTAA |
| *hmtC_Am_* | MT881545 | ATGTTCGTCCCTTCTCACTACCGCGAGCCAGACTCTTCTTGGATGGTCGATATCATCCGTGGCAACCCACTGGCCCTCATGATGTCTAACGGTGCTGCTGGCGAGCCTCCATTCGCTACCCACCTTCCTGTCATCCCCGACCCTGCTATGACTGGCGATTGGTCTGAGAGACTGTCTGAGGCCACCCTCCTTGGTCACATGAACCGCGATAACCCTCAGTGGCAGGCTTTGGAGGATGGTGCTGTCGTCAGAATCGCTTTCTCTGGTCCCCACGCTTACGTCTCTCCTACCCTTTACGGCGTCACTCCAGCTGCCCCCACCTGGAACTTCACTTCTGTCCACGTCCGTGGTGTCGTCGAGAGAATCCCTTCTACCGAGGAGACTCTTGAGGTCGTCAAGTCTACTGTCCGTGCTTTCGAGGCCGACTTCGGCGAGGGTTGGGATATGGCTGCCTCTATCGACTACTTCAGAAAGATCGTCCCTGGCGTCGGTGCCTTCCGCATCATGGTCCGTAACGTCGATGGCATGTTCAAGCTGTCTCAGGAGCAGCAACCAGAGGTCCGCGACCGTGTCAGAAAGTCTTTCGCTGGCAGAGAGTGCGGTCGCCACCAAGAGACCGCTGCCTACATGTCTCGTCTCCCATAA |
| *sfaC_Am_* | MT881549 | GTCTACGAGCGCCCTCTTTACCGTGAGGACTGCGATGGTGTCGTCCTCGCCTTCCTTCGCCACAACCCACTGGCTATGGTCGTCACTTCTCACGACGATGTCCCTGTCGCCACCCACGCTCCAGTCCTCTTCCGTCACGGTCCAGATGGTGCTGATGCTGAGGCTGTCGCTGCTGGTACTGTCCCACTGGCTGGTTCTACCCTCATCGGCCACATGAACGTCGAGAACCCTCAGTGGCGCCGTATGAGATCTGGCGACCGCGCTCTTATCGTCTTCCAAGGTCCCCACGGCTACGTCTCTCCTACCGTCTACGGTGTCACTCCCGCTGCCCCTACCTGGGACTTCATCGCTGTCCATGTCAACGGTACTGTCGAGCCAACTGCTGATCCCGCTGCCGTCCTCGACATCGTCTCTGATACTGCTAGACGCCTTGAGTCTGGCTTCGGTCGTGGCTGGGACCAGGAGTCTTCTCTCGATTACTTCAGACAAATCGCCCCTGGTGTCGGCGCTTTCACTCTGCGCGTCGATTCTGTCCAGACCATGTTCAAGCTCTCTCAAGAGAAGCCTGCCCCAATGCGTAGACGCGTCGTCGAGCAGTTCGAGGCTTCTGAGTCTGGCACCCACCGTGCCCTTGCTTCTGTCATGCGTGACAGAGGTCTGACTGAGGCCGATGAGGAGAGAGAGACCGCTGGCTAA |
| *padO_Am_* | MT881548 | ATGTTCGTCCCACAGCACTACAGAACTGATGATCGCCGTTGGCCTGTCCGTATCGTCCAAGACAACCCTCTCGCTCTCCTTATGTCTACCCGCGATGGCCGTGCTCCTTTCGCCTCTCACGTCCCAGTCATCGTCCTTCCCAGACAGCGCGAGGAGCTGGAGAGAACTGGCCGCTGGCAAGGTGCTGTCCTCCACGGTCACATGAACAGAGCCAACCCACACTGGAAGTCCTTGGCTGATGGTCAGCCTGCTGGTCTTGTCTTCCAAGGCCCTGCTGGTTACGTCTCTCCAGCCGTCTACAACACCTCTCCCGCTGTCCCTACTTGGAACTTCACTGCTGTCCATGTCCAGGGCCGCCTGAAGCTCGTCGCTGACGAGGAAGCTACTCTTGGTGTCGTCTCTGCTACTGCTAGACAGCTGGAGGAGAGATTCGGTGCTAGATGGACTGTCGAGCCTTCTGTCGATCACTTCCGTCAAATCCTGCCAGGCGTCGGTGCCTTCGAGCTCAGAGTCGAGGAGTGCGACTCTATGTTCAAGCTCTCTCAAGAGAAGGAGCACGAAGTCAGACACGCTGTCATGGATTGGTGCGCCCGCTCTCCCCGTGGTAGATCTAACGACCTTGCTGCCGTCATGCGTGATTACTACCCTCCAACCACTACCTGGCCTTCTTAA |
| *matF_Am_* | MT881546 | ATGTTCCGCCGTAGAGGTGTCACCCTTACTAAGGCTCTCCTTACCGCCGTCTGCATGCTGGCTGCCCCACTCACTCAGGCTATCTCTGTCGGCAACCTTACCTTCTCTCTGCCCTCTGAGACTGACTTCGTCTCTAAGCGTGTCGTCAACAACAACAAGTCTGCCAGAATCTACCGCATCGCTATCTCTGCCATCGACTCTCCAGGTTCTTCTGAGCTCAGAACTAGACCTGTCGATGGTGAACTGCTCTTCGCTCCTAGACAGCTCGCTCTTCAAGCCGGCGAGTCTGAGTACTTCAAGTTCTACTACCACGGCCCCAGAGATAACCGTGAGAGATACTACCGTGTCTCTTTCAGAGAGGTCCCAACCCGCAACCACACTCGCCGTTCTCCCACCGGCGGTGTCGTCTCTACTGAGCCTGTCGTCGTCATGGACACCATCCTGGTCGTCCGCCCTCGTCAGGTCCAATTCAAGTGGTCTTTCGATAAGGTCACCGGCACTGTCTCTAACACCGGTAACACTTGGTTCAAGCTTCTGATCAAGCCTGGCTGCGACTCTACTGAGGAAGAGGGCGATGCTTGGTACTTGCGCCCAGGCGATGTCGTCCACCAGCCCGAGCTCCGTCAACCTGGTAACCACTACCTTGTCTACAACGACAAGTTCATCAAGATCTCTGATTCTTGCCCAGCTAAGCCTCCATCTGCCGATTAA |
| *XF36_RS26795_Am_(XF36_Am_)* | MT881547 | ATGGTCGTCGGTATGACCGCTGAGACCTCTACTGTCCAGGGCGCTCTTTTCGGTGACCCTGCCGTCATCGAGACCACTGATCACGGCCCTACCGCCACTCAGGACCCACGTGAGGTCGCTAGAGTCGTCGGCCTTGCCCAAGATCCAGGTCTGTTCGTCGTCGAGAGAACTGGTCTTGTCCTGCGCGCTGATCCAGCTAGACCTGGTTGCGCTGATGCTCTGGCTCGTCACGACGGTGATACCGTCGCTCAACTCCTTGATACTGGCCACCTCAGACTTGGCGGTACCCACCATGTCCATCACAACGGTGATGAGGGTCCAGCTCGTTCTGTCCTCGTCCCTAAGGCCACTAGAGACATGGTCTCTCGCTGGGATCACCTTCGTCCTATCCCAGAGCCCACCAGAGCTCCTGAGCCAACTAAGGCCCCACAGAGATCTACTGGTCTCATCGGTGTCGATGTCGTCGAGCCTGGCAAGGCTCTGGTCTGCTTGGGTACCACTGGTCAAGGCGGTACTGTCCTGCGCGATGGCGGTAGATACCGCGTCGAGAACGACCATGGTGCTTTGGTCGGTCATGCTTCTTCTTACCGCGCTGCCGCTCGTCTGCTCGCTAGATACCACGGCTACGCCCCCGGTCCTGTCGAGATCGAGCACGAGCACCGTCTTCACCGCCGTTAA |

**Table S3.** Piperazate synthase genes from different hosts and their Genbank accession numbers

| Piperazate synthase genes | Producing Strain | Accession number |
| --- | --- | --- |
| *KtzT* | *Kutzneria sp.* 744 | EU074211 |
| *hmtC* | *Streptomyces himastatinicus* ATCC 53653 | FR823394 |
| *sfaC* | *Streptomyces flaveolus* DSM 9954 | FJ809786 |
| *padO* | *Streptomyces sp.* | KC915040 |
| *matF* | *Actinomadura atramentaria DSM 43919* | NZ_KB907224 |
| XF36_RS26795 | *Pseudonocardia sp.* HH130629-09 | CP011868 |

**Table S4.** DNA sequence for the promoter of phosphoglycerate kinase gene amplified from genomic DNA of *A. melanogenum* HN6.2 strain.

| Item | DNA sequence |
| --- | --- |
| *P_pgk_* | ATTACCTTCAAGCATCCATCGGACAGCGGGACAGGCAGGCGTTCTGGCAACTTCACGGGGCGTTACGGGCCGATCGATAGACATGTCGTGTGGTGCAAGGCATCTTGAATAACAAAGTGATCAAGATGAGCAACTTGAAGAAGGACACATGGTGCGATTTCGAGTGAAGCAGGTGATCCAAGGGCAGTTTGCATTTAGAGATGGGTCATGATTGGTGATATCAGTTGATGGGCAAACACGAGAGGGCACGAAGATCTCGGTAGCACGTCGGTGACGCGGTTTTTTGAGCTGCCCCTCATGCCGAGCGTCGGAAGCTGTTGGGGAAAGATTCAAGCCCCTCACACACCACAACAACCCATCAAGCTCTGTTGTATATAACTCTGTGCGTCTTTCTCTGTTGCTGTCGTTCACATCAACCTGTCTAACACATCCAATTCAGTCACA |

**Experimental procedures**

**Medium**

All the strains of *A. melanogenum* were routinely propagated at 28°C in YPD medium. The *E. coli* DH5α strain used for cloning and plasmid propagation was grown at 37°C in Luria–Bertani (LB) medium supplemented with 50.0 mg mL^-1^ of ampicillin. The media for yeast transformation and selection and the iron-depleted MSP medium were prepared as described previously (Lu*, et al.*, 2019). The iron-replete CM was composed of 3.73% (w/v) glucose, 1.40% peptone, 0.30% K_2_HPO_4_, 0.075% citrate monohydrate, 0.008% MgSO_4_, 0.0002% ZnSO_4_, and 0.32% YNB (without amino acids).

**Genetic manipulation**

Gene deletion and overexpression were implemented using the Cre–*lox* system following the methods described previously (Lu*, et al.*, 2019); the plasmid of pAMEXlox-2 was used for overexpressing genes. In particular, the native L-ornithine-N^5^-hydroxylase gene *sidA* was amplified from the genome of the *A. melanogenum* wild-type strain HN6.2, which was the origin of the DOLC strain used in this study. The heterologous piperazate synthase genes (Table S1) were artificially synthesized with codons optimized for *A. pullulans* (http://www.kazusa.or.jp/codon/cgi-bin/showcodon.cgi?spe-cies=5580&aa=1&style=N). Their sequences were listed in Table S2. The genetic cascade of *sidA*-*ktzT_Am_* was constructed by tandemly connecting the gene fragments of *sidA*, cyc1, and the promoter of phosphoglycerate kinase gene from the HN6.2 strain (*Ppgk*), *ktzT_Am_* and *cyc1* (*sidA-cyc1-Ppgk-ktzT_Am_-cyc1*) using overlap extension polymerase chain reaction (PCR). The obtained fragment was then integrated in the multiple cloning sites of pAMEXlox-2. *Ppgk* was amplified from the genome of the HN6.2 strain, the sequence of which is listed in Table S4.

Analytical and quantification methods

The analysis and quantification of extracellular siderophores and the quantification of intracellular L-ornithine content and dry cell weight were performed as discussed previously (Lu*, et al.*, 2019). Pre-column derivatization of extracellular fungal product with Fmoc-Cl and L-FDAA was performed in accordance with the methods described by Du and coworkers (Du*, et al.*, 2017). Fmoc-Cl derivatives were tested using a HPLC 1260 apparatus (Agilent, USA) with an Agilent ZORBAX Eclipse Plus C18 column (5 µm; 4.6 mm × 250 mm). Elution was performed at 1.0 mL min^-1^ with a mobile phase composed of a linear gradient of water and acetonitrile [(v/v): 60:40 to 0:100, 0–25 min], both of which contain 0.05% (v/v) trifluoroacetic acid. The detection wavelength was measured at 280 nm, and the column temperature was maintained at 30°C. For L-FDAA derivatives, elution was performed at 0.5 mL min^-1^ with a mobile phase composed of a linear gradient of water and acetonitrile [(v/v) 70:30, 0–3 min; 70:30–30:70, 3–18 min; 0:100, 18–22 min). The remaining procedures were identical to those described above. Quantification using LC–MS was performed (with the same column and elution conditions) on a 6120 Quadrupole LC-MS system (Agilent, USA) operated in selected ion monitoring mode, and *m/z* values of [M+H]^+^ ions for the amino acids derivatives above were monitored. A L/D-piperazic acid racemic standard as an external reference was purchased from Synchem OHG (Germany; purity = 95%) and dissolved in pure water to the final concentration of 1.0 mg mL^-1^ as a stocking solution. To quantify L-piperazic acid, a serious of standard L-piperazic acid solutions were first prepared by diluting the stocking solution into 50, 100, 200, 300, 400, 500, 1000 mg L^-1^, respectively. Each of them was subjected to Fmoc-Cl pre-column derivatization and the product was loaded into HPLC analysis, as described previously. The obtained peak areas were used to plot a calibration curves of L-piperazic acid against their peak areas from HPLC (Fig. S8a). With it, a tested peak area from HPLC could be substituted into the equation and a corresponding concentration of L-piperazic acid could be calculated.

Quantification of N^5^-hydroxy-L-ornithine was as well achieved using pre-column derivatization HPLC. First, the strains were pre-cultured for 2 days in 5.0 mL aliquots and used to inoculate 50.0 mL flask cultures in CM and allowed to cultivate for 36 h. Then, 1 mL of the culture was sampled and centrifuged to collect the cells for each strain. The supernatant from the cell lysate of each strain was prepared following the procedures of L-ornithine determination. The content of N^5^-hydroxy-L-ornithine in the supernatant was determined using Fmoc-Cl derivatization HPLC. The supernatant was filtered using a 0.22 μm syringe filter. The samples were then pre-column derivate with Fmoc-Cl, as described previously. Fmoc-Cl derivatives were tested using a HPLC 1260 apparatus (Agilent, USA) with Agilent ZORBAX Eclipse Plus C18 column (5 µm, 4.6 mm × 250 mm). Elution was performed at 1.0 mL min^-1^ using a mobile phase composed of a linear gradient of water and acetonitrile [(v/v): 60:40–0:100, 0–25 min], both of which contain 0.05% (v/v) trifluoroacetic acid. The detection wavelength was measured at 254 nm, and the column temperature was maintained at 30°C. A N^5^-hydroxy-L-ornithine standard as an external reference was purchased from Synchem OHG (Germany; purity = 98%) and dissolved in pure water to the final concentration of 1.0 mg mL^-1^ as a stocking solution. To quantify N^5^-hydroxy-L-ornithine, a serious of standard solutions were prepared by diluting the stocking solution into 10, 20, 30 40, 50 mg L^-1^, respectively. Each of them was subjected to Fmoc-Cl pre-column derivatization and the product was loaded into HPLC analysis, as described previously. The obtained peak areas were used to plot a calibration curves of N^5^-hydroxy-L-ornithine against their peak areas from HPLC (Fig. S8b). With it, a tested peak area from HPLC could be substituted into the equation and a corresponding concentration of N^5^-hydroxy-L-ornithine could be calculated. For the intracellular content of N^5^-hydroxy-L-ornithine, it was defined as milligram of N^5^-hydroxy-L-ornithine per milligram of intracellular total proteins determined by Bradford protein assay kit (Bio-rad, USA).

NMR analysis of the extracellular product, which had a retention time close to that of the L/D-piperazic acid standard in the Fmoc-Cl pre-column derivatization HPLC, was used to specify its structure and confirm it was indeed piperazic acid. Before NMR analysis, Fmoc-Cl derivatized extracellular products were purified using the LC-6ADVP preparative HPLC apparatus (Shimadzu, Japan). The purified products were checked again using HPLC as above and dehydrated by vacuum drying. The obtained powder was dissolved in a solution of 20% piperidine in dimethylformamide, followed by the addition of NaN_3_ to remove Fmoc moieties using the method described by Chen and coworkers (Chen*, et al.*, 2014). The resulting product was dehydrated by vacuum drying to obtain 10 mg of powder sample. This powder was dissolved in 1 mL of DMSO-d6 for 1D NMR, the data were collected using a Bruker 600MHz spectrometer (AVANCE NEO, Bruker, Germany).

Protein expression, purification, and specific enzyme activity

Codon-optimized genes were synthesized with fusion to 6× His tags, cloned into pET30a using NdeI and HindIII restriction sites, and transformed into *E. coli* BL21(DE3) for protein expression. Expression and purification of 6× His-tagged enzymes were performed as follows: the culture was grown at 37°C in LB medium in a baffled flask at 200 rpm with an appropriate antibiotic to an optical density of 0.5 AU at 600 nm. Isopropyl β-d-1-thiogalactopyranoside was added to a final concentration of 500 mM and the temperature was lowered to 16°C. After 10 h of further incubation, the cells were harvested and resuspended in lysis buffer (300 mM NaCl, 50 mM Tris–HCl, and 10 mM imidazole; pH 8.0) and then disrupted by sonication on ice, and the supernatant was recovered by centrifugation (13,000× *g* for 40 min). His-tagged protein was separated using nickel-nitrilotriacetic acid resin. After rinsing with washing buffer (300 mM NaCl, 50 mM Tris–HCl, and 50 mM imidazole; pH 8.0), the protein was eluted with elution buffer (300 mM NaCl, 50 mM Tris–HCl, and 250 mM imidazole; pH 8.0). Purified protein-containing fractions were confirmed by sodium dodecyl sulfate–polyacrylamide gel electrophoresis and then dialyzed into the storage buffer (50 mM Tris–HCl, 100 mM NaCl, and 10% glycerol; pH 8.0). The dialyzed protein was concentrated and stored at −80°C until further use. The protein concentration was determined using the Bradford protein assay kit (Bio-Rad, USA); bovine serum albumin was used to generate a standard curve.

The enzyme activity assay was conducted at 28°C in a 100-ml reaction volume containing 100 nM L-Piz synthetase in 50-mM Tris–HCl buffer (pH 8.0; containing 10 mM sodium dithionite). The reactions were preincubated at 28°C for 5 min before the addition of N^5^-hydroxy-L-ornithine in the final concentration of 1 mM to initiate the reaction. Each reaction condition was run in triplicates. The reactions were quenched at 5 min with 2 volumes of cold acetonitrile and stored at −80°C before Fmoc-Cl derivatization and HPLC analysis. Production of L-Piz was calculated based on a standard curve of L-Piz that was constructed under the same conditions as plotted above. One unit of enzyme activity was defined as the amount of enzyme required to catalyze the production of 1 mmol L-Piz per minute at 28°C. Specific enzyme activity was defined as the units of enzyme activity per milligram of protein.

qPCR

Quantitative PCR (qPCR) was used to determine the relative transcriptional levels of the purposed genes. RNA was extracted from mid-log phase cells using the RNA prep Pure Tissue Kit (TIANGEN, China), and cDNA was prepared using the Prime Script RT Reagent Kit (TaKaRa, Japan). The β-actin gene was used as the internal control. Relative transcription levels were calculated as described previously (Lu*, et al.*, 2019).

10.0-L fermentation

The strain DFAK1 was aerobically grown in YPD medium at 28.0°C for 48 h. Three hundred milliliters of the seed culture were transferred to 7.0 L of CM in a 10.0-L bioreactor (BIOQ-6005-6010B, Huihetang Bioengineering Equipment, Shanghai, China). The fermentation was performed under the following conditions: agitation speed, 300 rpm; aeration rate, 6.5 L min^-1^; temperature, 28°C; and fermentation duration, 120 h. For the 10.0-L fed-batch fermentation, 120.0 g of glucose was supplemented into the bioreactor at the 60^th^ hour of the fermentation. During the fermentation, 50.0 mL of the culture was harvested in intervals of 12 h and centrifuged at 12000*×* *g* for 10 min. The titer of L-piperazic acid in the obtained supernatant was measured as described above, and the content for the residual glucose in the supernatant was determined using a glucose assay kit (glucose oxidase method; Nanjing Jiancheng Bioengineering Institute, China). The dry cell weight in the centrifuged sediment was measured as described by us previously (Lu*, et al.*, 2019).

Statistical analysis

Statistical analysis was performed using Design Expert 7 (Stat-Ease, Minneapolis, MN, USA) statistical package. The results are reported as the means ± standard deviations for parametric data (n = 3). Analysis of variance and comparison of the means were checked for using Tukey's test. p < 0.05 was considered statistically significant.

**References**

Chagarovskiy, A.O., Ivanova, O.А., Shumsky, A.N., and Trushkov, I.V. (2017) Synthesis of hexahydropyridazin-3-ones by reactions between donor-acceptor cyclopropanes and phenylhydrazine, *Chem Heterocycl Com*+ **53**: 1220-1227.

Chen, C.C., Rajagopal, B., Liu, X.Y., Chen, K.L., Tyan, Y.C., Lin, F., *et al.* (2014) A mild removal of Fmoc group using sodium azide. *Amino Acids* **46**: 367-374.

Du, Y.L., Dalisay, D.S., Andersen, R.J., and Ryan, K.S. (2013) N-carbamoylation of 2,4-diaminobutyrate reroutes the outcome in padanamide biosynthesis. *Chem Biol* **20**: 1002-1011.

Lu, Y., Wang, H., Wang, Z., Cong, Y., Zhang, P., Liu, G., *et al.* (2019) Metabolic rewiring improves the production of the fungal active targeting molecule fusarinine C. *ACS Synth Biol* **8**: 1755-1765.
